# CXCL11 reprograms M2-biased macrophage polarization to alleviate pulmonary fibrosis in mice

**DOI:** 10.1186/s13578-024-01320-7

**Published:** 2024-11-15

**Authors:** Ji-Young Kim, Dong-Wook Cho, Jung-Yun Choi, Suji Jeong, Minje Kang, Woo Jin Kim, In-Sun Hong, Haengseok Song, Heesoon Chang, Se-Ran Yang, Seung-Joon Lee, Mira Park, Seok-Ho Hong

**Affiliations:** 1 Department of Internal Medicine, School of Medicine, Kangwon National University, Chuncheon, Republic of Korea; 2 Department of Health Sciences and Technology, GAIHST, Gachon University, Incheon, Republic of Korea; 3 Department of Biomedical Science, CHA University, Seongnam, Gyeonggi Republic of Korea; 4 KW-Bio Co., Ltd, Chuncheon, Republic of Korea; 5 Department of Thoracic and Cardiovascular Surgery, School of Medicine, Kangwon National University, Chuncheon, Republic of Korea

**Keywords:** Pulmonary fibrosis, Macrophage, Polarization, CXCL11

## Abstract

**Background:**

In understanding the pathophysiology of pulmonary fibrosis (PF), macrophage plasticity has been implicated with a crucial role in the fibrogenic process. Growing evidence indicates that accumulation of M2 macrophages correlates with the progression of PF, suggesting that targeted modulation of molecules that influence M2 macrophage polarization could be a promising therapeutic approach for PF. Here, we demonstrated a decisive role of C-X-C motif chemokine ligand 11 (CXCL11) in driving M1 macrophage polarization to alleviate PF in the bleomycin-induced murine model.

**Results:**

We intravenously administered secretome derived from naïve (M0) and polarized macrophages (M1 and M2) into PF mice and found that lung fibrosis was effectively reversed in only the M1-treated group, with modulation of the M1/M2 ratio toward the ratio of the control group. These findings suggest that the factors secreted from M1 macrophages contribute to alleviating PF by targeting macrophages and reshaping the immunofibrotic environment in a paracrine manner. Secretome analysis of macrophages identified CXCL11 as an M1-specific chemokine, and administration of recombinant CXCL11 effectively improved fibrosis with the reduction of M2 macrophages in vivo. Furthermore, a mechanistic in vitro study revealed that CXCL11 reprogrammed macrophages from M2 to M1 through the activation of pERK, pAKT, and p65 signaling.

**Conclusions:**

Collectively, we demonstrate an unprecedented role for M1 macrophage-derived CXCL11 as an inducer of M1 macrophage polarization to revert the fibrogenic process in mice with PF, which may provide a clinically meaningful benefit.

**Supplementary Information:**

The online version contains supplementary material available at 10.1186/s13578-024-01320-7.

## Introduction

Macrophages are highly plastic cells, the functions and phenotypes of which are modulated according to the local microenvironment. Macrophages, the most abundant immune cell type in healthy lungs, exert key functions in the homeostasis of lung tissue but are also involved in the pathogenesis of lung diseases, including pulmonary fibrosis (PF) [1]. During the early inflammatory phase of PF, activation of M1 macrophages promotes inflammation through the secretion of pro-inflammatory cytokines and matrix metalloproteinases, and this sustained inflammatory response acts as an important trigger to initiate a fibrotic response. During the lung repair process, M2 macrophage polarization is enhanced in the presence of interleukin-4 (IL-4) and IL-13, and their predominant infiltration serves as a critical regulator of the development and progression of PF, with the excessive accumulation of collagen and extracellular matrix (ECM). A higher proportion of anti-inflammatory M2 macrophages was observed in bronchoalveolar lavage fluid from human patients with idiopathic PF (IPF) and in mouse lung tissue in bleomycin (BLM)-induced PF model [2–5]. Thus, suppression of M2 macrophage polarization, macrophage repolarization from M2 to M1, and promotion of M2 macrophage apoptosis have been suggested as promising strategies for the treatment of PF. However, no clinically applicable therapy currently targets M2 macrophages to suppress PF. In addition, it is still not fully understood how macrophage polarization affects the function of alveolar epithelial cells (AECs) and fibroblasts in the lung in steady and diseased states.

Chemokines largely regulate macrophage trafficking to sites of injury. Chemokines (chemotactic cytokines 8–10 kDa in size) are classified into four subfamilies, CC, CXC, CX3C, and XC, according to the composition of the two cysteine residues closest to their N termini, and bind to receptors selectively expressed on the surface of target cells to directly induce cell activation processes, such as cell migration and proliferation [6, 7]. As key mediators linking inflammation and fibrogenesis, chemokines induce fibroblast activation, contributing to the formation of a fibrotic microenvironment [8]. More importantly, studies have focused on their roles in modulating macrophage polarization during pulmonary injury. Chemokine C–C motif ligand 1 (CCL1) and CCL2 attract immune cells to the lungs and induce M2 macrophage activation in a CCL1/CC chemokine receptor 8 (CCR8) axis [9]. In addition, prolonged exposure to the anti-inflammatory cytokine IL-10 activates M2 macrophages in lung tissue and activates myofibroblasts through upregulation of the CCL2/CCR2 axis, leading to the formation of a fibrotic microenvironment [10]. Moreover, a CCL17/CCR4-dependent increase in M1 macrophages during the acute inflammatory phase was shown in BLM-induced lung tissues, leading to lethal inflammatory and fibrotic responses [11]. However, such responses were not seen in CCR4^−/−^ mice, and M2 phenotypes significantly increased during the peak of pulmonary inflammation. The selective blockade of the expression of specific chemokines and their receptors may be an effective way to inhibit PF. Targeted activation or stabilization has also been shown to have a beneficial role. C-X-C chemokine receptor 3 (CXCR3)-deficient mice showed severe BLM-induced lung injury and developed progressive interstitial fibrosis [12]. In vitro, epithelial-to-mesenchyme transition induced by transforming growth factor β1 (TGF-β1) was inhibited by CXCL9 [13]. CXCL11 prevented the development of BLM-induced lung fibrosis via the repression of aberrant vascular remodeling [14]. These findings highlight the importance of the chemokine-mediated regulation of macrophage function during fibrotic remodeling [15]. Elucidating the complex relationship between anti-fibrotic chemokines and receptor regulation may provide new therapeutic approaches to halt or even reverse the progression from chronic inflammation to fibrosis.

In this study, we aimed to investigate whether the reprogramming of pro-fibrotic macrophages to anti-fibrotic macrophages leads to a subsequent reduction in fibrosis. By assessing the kinetics of macrophage polarization in vivo during PF pathogenesis, we obtained data revealing a disproportionate bias in the macrophage phenotype due to excessive M2 macrophage infiltration. We screened for modulators that can convert macrophages from the M2 to the M1 phenotype and investigated their anti-fibrotic properties. CXCL11, which is secreted explicitly by anti-fibrotic macrophages, showed effective anti-fibrotic properties by targeting M2 macrophages and reprogramming them to an M1 phenotype, providing new possibilities for macrophage-based therapeutic strategies.

## Results

### Predominant accumulation of M2 macrophages during the fibrotic progression phase in BLM-induced mouse lung tissues

**Fig. 1:**
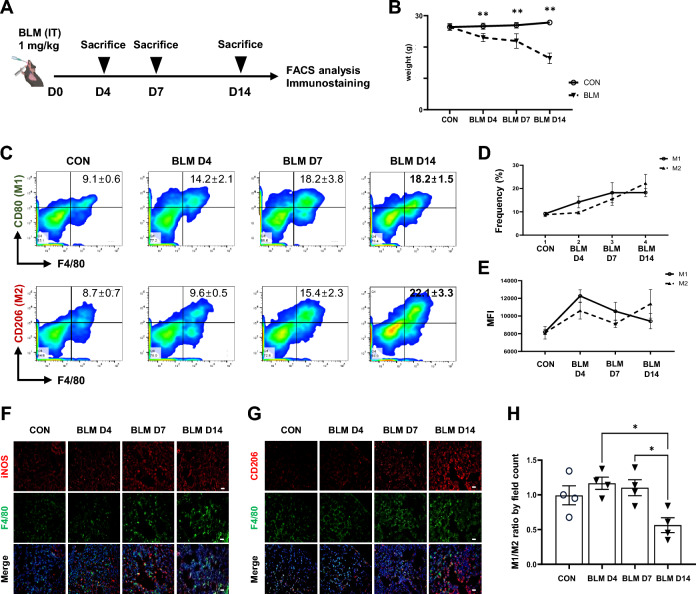
Polarization of macrophages during the phase of fibrotic progression in BLM-induced mice lung tissues. **A** Schematic overview of study BLM-induced PF mice model. **B** The body weight of the mice was measured from the start of BLM administration to the time of sacrifice (days 4, 7, and 14). **C, D** Flow cytometry analysis of the expression of M1 (F4/80+ iNOS+) and M2 (F4/80+ CD206+) macrophages in lung tissues of BLM-induced PF mice. **E** Mean fluorescence intensity (MFI) of M1 and M2 markers. **F, G** Representative images of immunostaining for iNOS (**F**) CD206 (**G**) and F4/80 in the lung tissues of each group. Scale bars, 20 μm. **H** The ratio of M1 and M2 macrophages was analyzed by counting the number of M1 and M2 positive cells in at least three different fields of lung tissues of each group. Data are presented as mean ± SD. *P < 0.05, **P < 0.01

As macrophages exhibit distinct functional phenotypes depending on their microenvironment, we assessed the in vivo kinetics of macrophage polarization in a BLM-treated PF mouse model. Intratracheal BLM was administered to mice, and lung macrophage phenotypes were analyzed by flow cytometry on days 4, 7, and 14 after treatment (Fig. 1A). Due to the toxicity of BLM, a time-dependent weight loss was observed in the PF model (Fig. 1B). BLM-treated mice showed increased macrophage infiltration in the lung during the inflammatory phase from day 0 to day 7. As expected, the frequency of M1 macrophages (F4/80^+^CD80^+^, 14.2 ± 2.1%) was higher compared to that of M2 macrophages (F4/80^+^CD206^+^, 9.6 ± 0.5%) during the inflammatory phase. In contrast, M2 macrophages were detected more predominantly than M1 macrophages at day 14 (M2, 22.4 ± 3.3% vs. M1, 18.2 ± 1.5%), indicating a shift from anti-fibrotic M1 to pro-fibrotic M2 macrophages in the fibrotic phase of BLM-induced PF in mice (Fig. 1C, D). This shift was also clearly evident in the measurement of mean fluorescence intensity (MFI) and upon immunostaining for M1 and M2 markers, respectively (Fig. 1E–H). These findings suggest that macrophage recruitment to the lung is enhanced in the inflammatory phase of BLM-induced PF, and as inflammation persists, a transition to a fibrotic microenvironment rich in M2 macrophages occurs.

### M1 macrophages attenuate BLM-induced lung damage in a paracrine manner

**Fig. 2:**
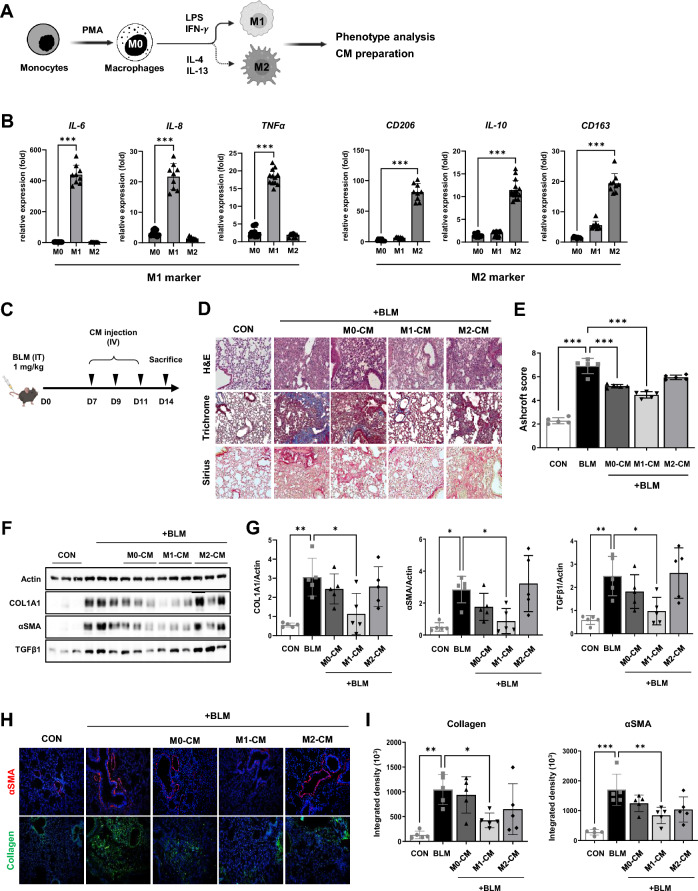
Administration of M1-CM reverts BLM-induced PF in mice. **A** Schematic overview of macrophage differentiation and activation. **B** The expression levels of *IL6*, *IL8*, and *TNFα* (M1 markers) *and CD206*, *IL10,* and *CD163* (M2 markers) were validated by qPCR. Data are presented as mean ± SD. **C** Schematic overview for testing the efficacy of the CM collected from naïve and polarized macrophage in the PF mice model. **D** Representative images of hematoxylin and eosin (HE), Masson’s trichrome, and Sirius Red staining in lung tissue from each group. Scale bars, 50 μm. **E** The Ashcroft score indicates the severity of fibrosis. **F** Western blotting for COL1A1, αSMA, TGFβ1, and Actin as a loading control in the lung homogenates of each group. **G** The graph shows the relative intensity of COL1A1, αSMA, and TGFβ1 in the lung tissue of the indicated groups. **H** Representative images of immunostaining for αSMA and Collagen in the lung tissue from each group. Scale bars, 50 μm. **I** Quantification of Collagen and αSMA positive areas by Image J 1.51j. Data are presented as mean ± SD. *P < 0.05, **P < 0.01., ***P < 0.001

Given the important role of the M2 macrophage-rich lung microenvironment in the development of PF, we first examined whether fibrotic lung tissues can be repaired by secretions from different types of macrophages. To evaluate the therapeutic potential of macrophages, monocytes were differentiated into macrophages and polarized with either lipopolysaccharide (LPS) and interferon (IFN)-γ (M1) or IL-4 and IL-13 (M2) (Fig. 2A). Macrophage polarization was confirmed by examining the expression levels of M1 (*IL6*, *IL8*, and *tumor necrosis factor α* (*TNFα*))- and M2 (*CD206*, *IL-10*, and *CD163*)-specific genes (Fig. 2B). We intravenously (IV) administered conditioned media (CM) collected from naïve (M0) and polarized (M1 and M2) macrophage cultures to mice on days 7, 9, and 11 after BLM induction and then examined histological and molecular markers of fibrosis (Fig. 2C). Hematoxylin and eosin (HE) staining revealed severe lung damage characterized by thickened alveolar septa and increased mononuclear infiltration in mice exposed to BLM. Extensive deposition of fibrillar collagen was also clearly observed in the lung tissues of fibrosis mice by Masson’s trichrome and Sirius Red staining (Fig. 2D). These fibrosis symptoms were markedly improved in the lung tissue of M1–CM-treated mice compared to those of M0– and M2–CM-treated mice (Fig. 2D). The Ashcroft scores were significantly lower in the M0– and M1–CM-treated groups than in the BLM-treated group (Fig. 2E). Similar to the histological analysis, only M1–CM treatment significantly reduced the protein levels of fibrosis-related factors (α-smooth muscle actin (αSMA), collagen, type 1, alpha 1 (COL1A1), and TGF-β1) compared to M0–CM and M2–CM treatments, as determined by Western blot (Fig. 2F, G) and immunofluorescence staining (Fig. 2H, I). These results suggest the paracrine, anti-fibrotic effects of M1-polarized macrophages on the impaired lung during the phase of fibrotic progression in BLM-treated mice.

### The anti-fibrotic activity of M1–CM is accompanied by enhanced type 2 AEC proliferation and an M2-to-M1 macrophage transition

**Fig. 3:**
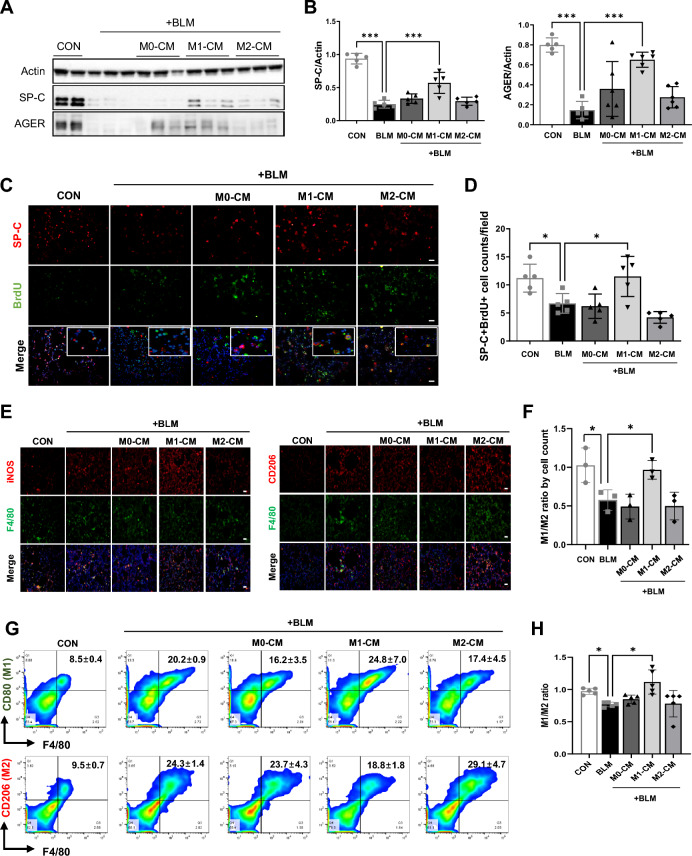
M1 macrophage-CM promotes proliferation of AECs. **A, B** Western blotting (**A**) and subsequent quantification of SP-C (type 2 AEC marker) and AGER (type 1 AEC marker) (**B**) in whole-lung homogenates of mice from the indicated groups. Actin was used as a loading control. **C** Representative images of immunostaining for colocalization of BrdU and SP-C from lung tissues from each experimental group. Scale bars, 20 μm. **D** The SP-C + BrdU + cells were counted in at least 3 different fields of lung tissues of each group. **E** Representative images of immunostaining for iNOS, CD206, and F4/80 in the lung tissues of each group. Scale bars, 20 μm. **F** The ratio of M1 and M2 macrophages was analyzed by counting the number of M1 and M2 positive cells in at least three different fields of lung tissues of each group. **G** Flow cytometry analysis of the expression of M1 (F4/80+ CD80+) and M2 (F4/80+ CD206+) macrophages in lung tissues from each group. **H** Flow cytometry analysis of the M1/M2 ratio in each group. Data are presented as mean ± SD. *P < 0.05, ***P < 0.001

After lung injury, dysfunctional macrophages driven by unregulated inflammatory mediators were found to disrupt AEC differentiation and impair alveolar regeneration [16, 17]. Therefore, we next asked whether M1–CM, with its anti-fibrotic activity, is capable of promoting alveolar regeneration in the lung tissue of BLM-treated mice. Western blot analysis revealed that the marked reductions of surfactant protein C (SP-C, type II AEC marker) and advanced glycosylation end-product receptor (AGER, type I AEC marker) observed in the lung tissue of PF mice were significantly recovered in the M1–CM-treated group but not in the M0– and M2–CM-treated groups (Fig. 3A, B). Furthermore, the percentage of SP-C^+^ cells co-stained with bromodeoxyuridine (BrdU) was significantly increased in lung tissues of the M1–CM-treated group compared to the BLM-treated group, whereas M0– and M2–CM treatments did not affect the proliferation of type II AECs in fibrotic lung tissues (Fig. 3C, D). These findings suggest that M1 macrophages could not only suppress the features of lung fibrosis but may also contribute to the regeneration of impaired AECs. Previous studies have reported that the balance between M1 and M2 macrophages is a key factor in the pathogenesis of PF [18]. Thus, we further evaluated the effects of M1–CM on macrophage polarization in the lung tissues of PF mice by measuring the numbers of M1 and M2 macrophages (inducible nitric oxide synthase (iNOS)^+^F4/80^+^ and CD206^+^F4/80^+^, respectively) using immunostaining (Fig. 3E, F). As shown in Fig. 3F, the M1/M2 ratio was significantly decreased in the BLM-treated group compared to the control group. Interestingly, the M1/M2 ratio returned to normal in the M1–CM-treated group but not in the M0– and M2–treated groups. We also analyzed the polarization of lung macrophages by flow cytometry and showed that the M1/M2 ratio was restored to normal in the M1–CM-treated group (Fig. 3G, H). These results demonstrate that M1 macrophages could decrease pro-fibrotic M2 macrophage polarization in vivo in a paracrine manner. In addition, these findings suggest that regulating macrophage polarization might be a critical therapy to prevent or revert PF.

### M1 macrophage-derived CXCL11 induces the reprogramming of pro-fibrotic macrophages to anti-fibrotic macrophages in vitro

**Fig. 4:**
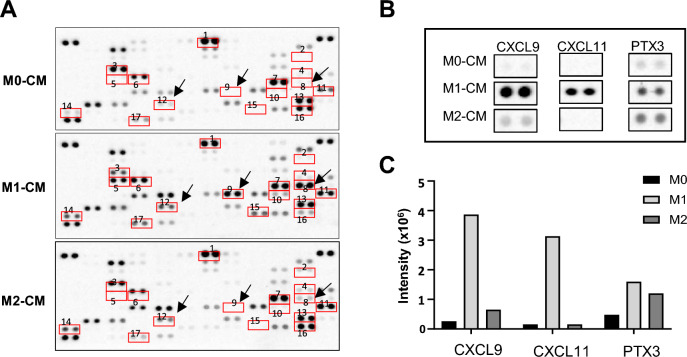
Cytokine analysis of CM from M0, M1, and M2 macrophages. **A** Comparative analysis of cytokine secretion in CM from naïve and polarized macrophage-CM. Each cytokine was detected in duplicate. Target cytokines are indicated using square frames with numbers. **B** CXCL9, CXCL11, and PTX3 proteins (indicated by arrows in a panel) were highly detected in M1-CM compared to M0- and M2-CM. **C** Quantification shows the mean signal intensity

**Fig. 5:**
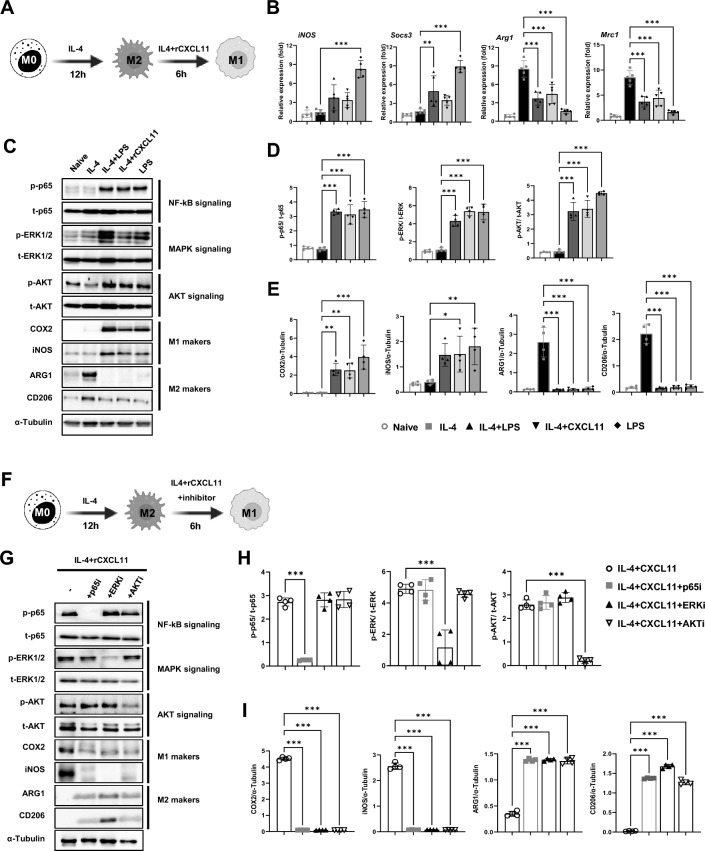
M1 macrophage-derived CXCL11 promotes M2 to M1 phenotype polarization mediated through the p65, ERK1/2, and AKT pathway in vitro*.* **A** A schematic diagram illustrating the experimental procedures to examine the effect of CXCL11 on macrophage polarity. **B** Real-time RT-PCR analyzed the relative mRNA levels of M1 (*iNos* and *Socs3*)- and M2 (*Arg1* and *Mrc1*)-related factors. **C** Western blotting for phosphorylation of p65, ERK1/2, AKT and COX2, iNOS, ARG1, and CD206 protein in BMDM after CXCL11 treatment. α-Tubulin was used as a loading control. **D, E** The graph shows the relative intensity of phosphorylation of p65, ERK1/2, and AKT (**D**) and COX2, iNOS, ARG1, and CD206 (**E**). **F** A schematic diagram illustrating the experimental procedures to examine the pathway of CXCL11 on macrophage polarity. **G** Western blotting for phosphorylation of p65, ERK1/2, AKT and COX2, iNOS, ARG1, and CD206 protein in BMDM after CXCL11 treatment with inhibitors. α-Tubulin was used as a loading control. **H, I** The graph shows the relative intensity of phosphorylation of p65, ERK1/2, and AKT (H) and COX2, iNOS, ARG1, and CD206 (**I**)

We have shown that a mediator of M2-to-M1 reprogramming during the pathogenesis of PF is CXCL11, which is secreted by anti-fibrotic macrophages (Fig. 4A–C). To determine if this effect is direct, we investigated the influence of CXCL11 on the reprogramming of macrophages ex vivo in a bone marrow-derived macrophage (BMDM) culture. BMDMs were treated with macrophage colony-stimulating factor (M-CSF) and IL-4 to differentiate into a pro-fibrotic macrophage (M2) phenotype, and the effects of CXCL11 on these M2 macrophages were analyzed (Fig. 5A). The LPS group was used as a positive control for M1 macrophages, the IL-4 group was used as a positive control for M2 macrophages, and the IL-4 plus LPS group was used as a control for the IL-4 plus rCXCL11 group. IL-4-induced macrophages showed significant increases in the expression levels of M2 markers (*Arg1* and *Mrc1*), while the expression levels of M1 markers (*iNOS* and *Socs3*) decreased, and co-treatment with IL-4 and CXCL11 resulted in increased expression of M1 markers (*iNOS* and *Socs3*) and decreased expression of M2 markers (*Arg1* and *Mrc1*), similar to the group co-treated with IL-4 and LPS (Fig. 5B). To identify the mechanisms underlying the M2-to-M1 phenotype change, the phosphorylation levels of mitogen-activated protein kinase (MAPK) and protein kinase B (AKT) were examined. We found that treatment of M2-differentiated macrophages with CXCL11 induced the phosphorylation of extracellular signal-related kinase (ERK), AKT, and p65 compared to IL-4 alone. We then further examined the protein expression levels of M1 markers (cyclooxygenase 2 (COX2) and iNOS) and M2 markers (arginase 1 (ARG1) and CD206) in response to phosphorylation signaling. M2 markers increased by IL-4 treatment were downregulated by CXCL11 and LPS treatment; in contrast, markers of M1 macrophages were significantly upregulated (Fig. 5C–E). Therefore, to determine whether pERK, pAKT, and p65 signaling are crucial for the determination of the M1 macrophage phenotype, inhibitors corresponding to each pathway were employed (Fig. 5F). Treatment of IL-4-treated M2 macrophages with the mitogen-activated protein kinase kinase (MEK) 1/2 inhibitor U0126, the AKT inhibitor wortmannin, and the p65 inhibitor JSH-23, respectively, suppressed CXCL11 treatment-induced phosphorylation, leading to the downregulation of M1 markers and the upregulation of M2 markers (Fig. 5G–I). These data support a direct effect of CXCL11 to induce the reprogramming of macrophages from M2 to M1.

### CXCL11 ameliorates PF through the modulation of macrophage phenotypes in vivo

**Fig. 6:**
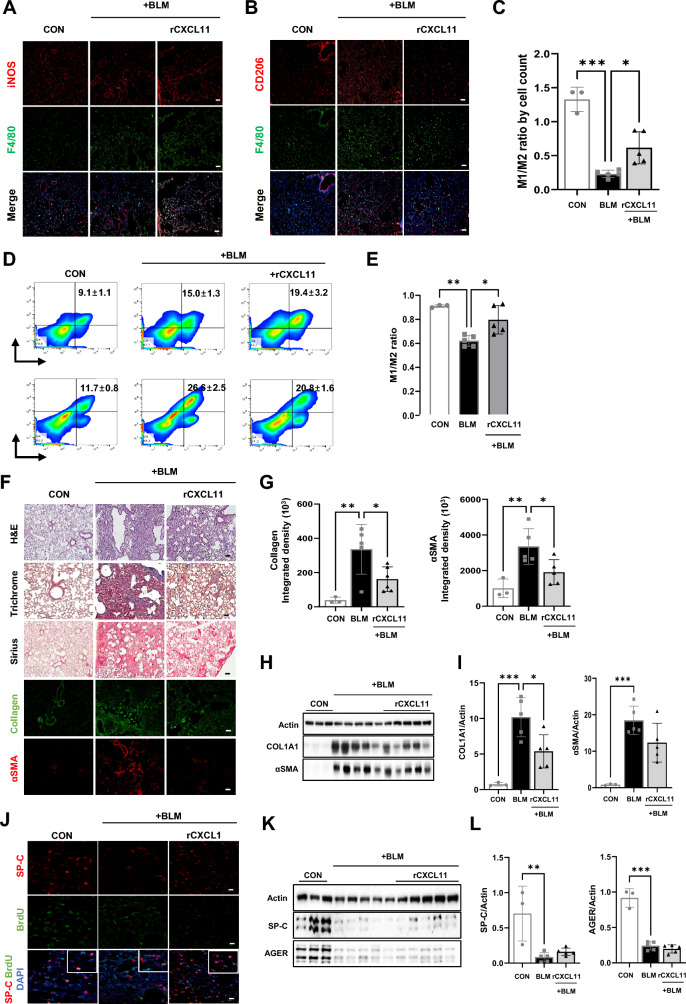
CXCL11 exhibits anti-fibrotic activity through the conversion of M2 macrophages to M1 macrophages. **A, B** Representative images of immunostaining for iNOS, CD206, and F4/80 in the lung tissue from each group. Scale bars, 20 μm. **C** The ratio of M1 and M2 macrophages was analyzed by counting the number of M1 and M2 positive cells in at least three different fields of lung tissues of each group. **D** Flow cytometry analysis of the expression of M1 (F4/80+ CD80+) and M2 (F4/80+ CD206+) macrophages in lung tissues from each group. **E** Flow cytometry analysis of the M1/M2 ratio in each group. **F** Representative images of HE, Masson’s trichrome, Sirius red staining, and immunostaining for Collagen and αSMA in lung tissue from each experimental group. Scale bars, 100 μm. **G** Quantification of Collagen and αSMA positive areas by Image J 1.51j. **H** Western blotting for COL1A1, αSMA, and Actin as a loading control in the lung homogenates of each group. **I** The graph shows the relative intensity of COL1A1 and αSMA in the lung tissue of the indicated groups. **J** Representative images of immunostaining for colocalization of BrdU and SP-C from lung tissues from each experimental group. Scale bars, 20 μm. **K** Western blot analysis of SP-C, AGER, and Actin as a loading control in the lung homogenates of each group of mice. **L** Subsequent quantification of SP-C and AGER in lung tissue of the indicated groups. Data are presented as mean ± SD. *P < 0.05, **P < 0.01., ***P < 0.001

Considering that the pro-fibrotic M2 phenotype was reprogrammed to the anti-fibrotic M1 phenotype upon CXCL11 treatment in vitro, it was reasonable to assume that CXCL11 could ameliorate BLM-induced PF in mice by modulating M2 macrophage polarization. Thus, we intravenously injected exogenous CXCL11 into mice during the fibrotic phase and then analyzed macrophage phenotypes in lung tissue by immunostaining. The imbalance in the M1/M2 ratio in the lungs of PF mice was significantly recovered by the infusion of CXCL11 (Fig. 6A–C). Furthermore, analysis of lung macrophages by flow cytometry showed that infusion of CXCL11 restored the M1/M2 ratio (Fig. 6D, E). However, not all M2 macrophages responded, indicating that CXCL11 alone may only partially affect M2 polarization and suggesting there is more to understand about the paracrine reprogramming effects of M1 on macrophage phenotypes. Furthermore, administering CXCL11 significantly improved tissue lesions, such as lung structure damage by BLM, and inhibited the deposition of collagen and αSMA (Fig. 6F–I). However, CXCL11 had no beneficial effects on the regeneration of impaired AECs in vivo (Fig. 6J–L). Taken together, our data highlight an unprecedented role for M1 macrophage-derived CXCL11 as an inducer of M1 macrophage polarization to revert the fibrogenic process in mice with PF, an effect that might be meaningful in clinical settings.

## Discussion

In this study, we explored the role of macrophage polarization in the progression and treatment of BLM-induced PF in mice. Our findings reveal a dynamic shift from anti-fibrotic M1 macrophages to pro-fibrotic M2 macrophages during the fibrotic progression phase, suggesting a pivotal role of macrophage phenotypes in fibrosis development. Notably, M1-polarized macrophages, through their secretome, significantly attenuate fibrosis markers, enhance lung tissue regeneration, and promote the proliferation of type II AECs. Additionally, M1-derived CXCL11 reprograms M2 macrophages into the M1 phenotype, restoring the M1/M2 balance and reducing fibrosis in vivo. Recently, Pouyanfard et al. compared the therapeutic efficacy of M1 and M2 macrophages derived from human induced pluripotent stem cells (hiPSCs) in a mouse model of liver fibrosis (LF) to answer the question of whether human-polarized macrophages can be used in the treatment of LF [19]. However, no studies have compared the anti-fibrotic properties of naïve and polarized human macrophages for their therapeutic effects on PF in vivo. In our study, for the first time, we tested the anti-fibrotic efficacy of naïve and polarized human macrophages in the fibrotic phase and showed the predominance of M2 macrophages in PF mice. We also demonstrated the therapeutic potential of M1–CM, which promoted M1 macrophage polarization and could indicate a key step toward the utilization of human-polarized macrophages in clinical therapeutic strategies for fibrotic diseases.

CXCL11 is primarily associated with M1 macrophages, promoting inflammatory responses and playing significant roles in the progression of cancer and fibrotic diseases. As a key chemokine that regulates macrophage polarization, CXCL11 influences inflammatory reactions and tissue regeneration, making it an important target in the treatment of inflammatory diseases and cancer [20–22]. Our study supports the idea that therapeutic interventions targeting macrophage polarization can modulate fibrotic outcomes. However, this effect was not as marked as the infusion of M1–CM, probably due to its synergy with other proteins, such as CXCL9 and pentraxin 3 (PTX3), which are abundantly detected in M1–CM. It has been shown that CXCL9 and PTX3 attenuate fibrotic changes and modulate macrophage polarization in the lung tissues of BLM-induced PF mice, suggesting interplay with macrophages during fibrogenesis [13, 23]. Furthermore, it is unclear whether the ontologically distinct tissue-resident macrophages, as opposed to the monocyte-derived macrophages studied in this research, play different roles in the pathological progression of fibrosis. Studies by Alexander et al*.* and McCubbrey et al*.* suggest that monocyte-derived macrophages, but not tissue-resident macrophages, are primarily responsible for the progression of fibrosis [24, 25]. Conversely, Osterholzer et al*.* highlighted Ly6C^hi^ monocyte-derived non-resident macrophages in the fibrosis process in a repeated injury model of type II alveolar epithelial cell-induced fibrosis, and Meziani et al*.* demonstrated that a population of tissue-infiltrating macrophages and alveolar macrophages are responsible for pulmonary fibrosis in radiation-induced lung fibrosis [26, 27]. Therefore, further studies are needed to compare the anti-fibrotic effects of single and combination treatments of CXCL11 with other M1-specific proteins or clinically approved PF drugs, such as pirfenidone and nintendanib, to achieve better therapeutic outcomes.

Numerous studies have provided evidence supporting significant roles for various external stimuli, including chemicals, small molecules, and microRNAs (miRs), in the treatment of PF by promoting the polarization of macrophages to the M1 phenotype or suppressing their polarization to the M2 phenotype [5, 28–30]. Questions have also been raised about whether the adoptive transfer of activated macrophages or macrophage-derived secretions, such as extracellular vesicles, could provide other avenues for the treatment of PF. Interestingly, exosomes purified from naïve or activated macrophages have been shown to attenuate fibrosis through the delivery of specific miRs or proteins in animal models [31, 32]. Our findings resonate with the regulatory role of miR-29c in ECM remodeling, as described by Xie et al. [33]. Although miR-29c suppresses the expression of fibrosis-related genes, our study demonstrates that the shift from M1 to M2 macrophages contributes to ECM deposition and fibrosis. This observation suggests that targeting macrophage polarization in conjunction with modulating miR-29c levels could offer a synergistic approach to controlling ECM remodeling and mitigating fibrosis in pulmonary conditions.

M1 macrophages suppress tumor growth due to intrinsic phagocytic and enhanced anti-tumor inflammatory reactions, suggesting that CXCL11 may have a tumor-resistant function in the tumor microenvironment (TME) repolarizing pro-tumor M2 macrophages into anti-tumor M1 macrophages. Contrary to our expectations, recent studies have shown that CXCL11 induces M2 polarization in the TME, promoting cancer growth. For example, the selective recruitment of CXCR3(+) B cells by pro-inflammatory IL-17 induced protumorigenic M2 macrophage polarization in human hepatocellular carcinoma [34]. CXC chemokines, particularly CXCL1, 8, 10, and 11, play roles in acute myeloid leukemia tumor processes [35]. Moreover, in clear cell renal cell carcinoma (ccRCC) tissue, RNA-binding motif protein 15 promoted M2 polarization by enhancing the stability of CXCL11 mRNA in an m6A-dependent manner, which resulted in ccRCC progression [36]. More recently, Wang et al. identified CXCL11 as a key factor in promoting the progression of multiple myeloma, which was positively correlated with M2 macrophage polarization in the TME [37]. The discrepancy in the function of CXCL11 on macrophage polarization between PF and the TME may be due to differences in the properties of normal and tumor-associated macrophages in terms of surface marker expression, secreted factors, and functions [38]. These findings indicate that CXCL11 may modulate macrophage polarization differently depending on the disease and organ type. Thus, before evaluating the therapeutic efficacy of CXCL11, it is critical to first understand the role of macrophage polarization in specific diseases and pathological conditions.

## Conclusion

**Fig. 7:**
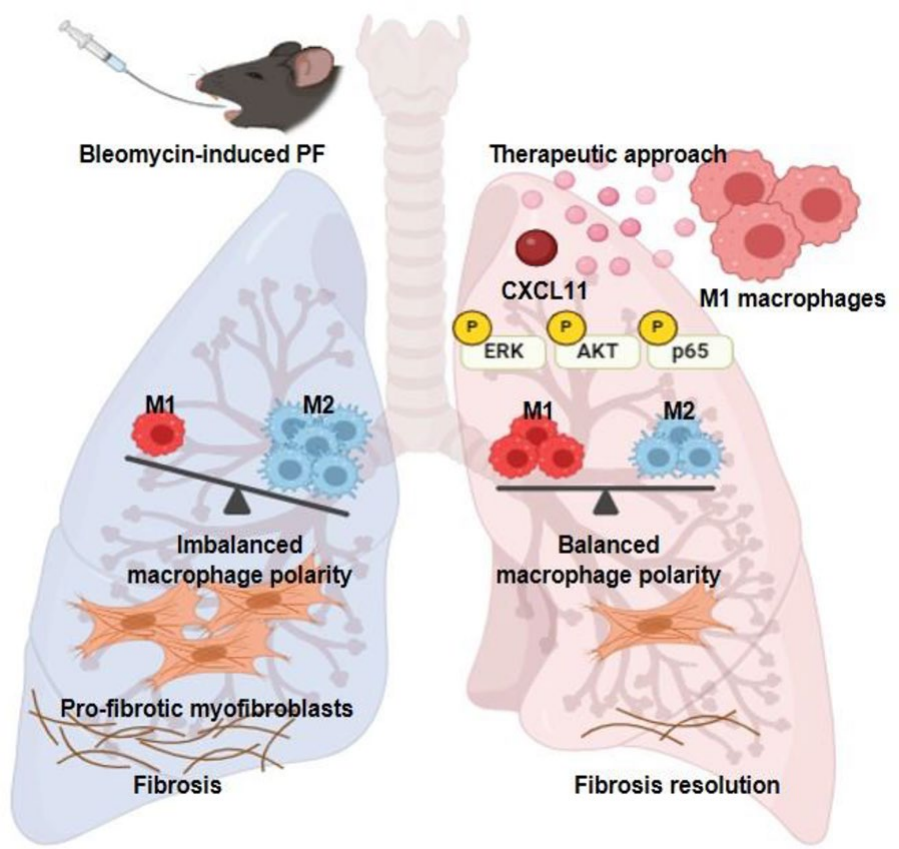
A schematic illustration of M1 macrophage-derived CXCL11 in BLM-induced PF in mice. M1 macrophage-derived CXCL11 to modulate M1 macrophage polarization for reverting the fibrogenic process in mice with PF

In summary, our results from this study provide evidence that human M1 macrophages improve PF in vivo through their secretion and newly define CXCL11 as the effective component in M1 secretion that can shift macrophage polarization from the M2 to the M1 phenotype and reduce fibrotic changes (Fig. 7). Alternatively, the adoptive transfer of primary and hiPSC-derived macrophages might be a promising approach for the treatment of PF [39]. However, the inherent plasticity of macrophages presents a significant challenge, as transplanted macrophages can alter their phenotype and function under pathological conditions such as oxidative stress [40, 41]. The dynamic switching between functional polarization and phenotypes of macrophages in response to the surrounding microenvironment can lead to unpredictable outcomes and necessitates cautious approach [42]. Therefore, genetically fixed M1 macrophages need to be established to overcome the uncertainty of phenotype duration and to provide more effective and safer cell therapies in the future.

## Material and methods

### Cell culture and macrophage differentiation

The immortalized human monocyte (hM) cell line was kindly provided by the Gachon University. hM cells were cultured in RPMI 1640 medium (Thermo, Waltham, MA, USA) supplemented with 10% heat-inactivated fetal bovine serum (Thermo). hM cells were seeded at a cell density of 1 × 10^6^ cells/60 mm dish and differentiated into naïve macrophages (M0) treated with 60 ng/ml phorbol 12-myristate 13-acetate (Sigma-Aldrich, St. Louis, MO, USA) for 48 h. The medium containing the PMA was then removed, and the dishes were washed with Dulbecco’s Phosphate Buffered Saline (DPBS). M0 macrophages were stimulated with lipopolysaccharide (Sigma-Aldrich) and interferon-gamma (20 ng/ml, Miltenyi Biotec, Bergisch Gladbach, Germany) for 48 h to induce M1 macrophages. M2 macrophages were obtained by incubation with IL-4 (20 ng/ml, Miltenyi Biotec) and IL-13 (20 ng/ml, Miltenyi Biotec) for 72 h. All cells were cultured in an incubator with 5% CO_2_ and at 37 °C.

### The preparation of human macrophage-derived conditioned medium (CM)

Human M0, M1, and M2 macrophages were seeded on 100-mm dishes (3 × 10^6^ cells/dish) and incubated with serum-free RPMI1640 for 24 h at 37 °C in a humidified atmosphere with 5% CO_2_. The CM was collected, filtered through a 0.22 μm filter, and concentrated with a 3-kDa cutoff ultrafiltration membrane (MilliporeSigma, Burlington, MA, USA) using an ultracentrifuge at 4 °C and 6000×*g* for 1 h.

### BLM-induced PF model

Male C57BL/6J mice (23–25 g, 10–12 weeks old) were purchased from DooYeol Biotch (Seocho, Seoul, Korea), and all animal experiments were approved by the Institutional Animal Care and Use Committee of Kangwon National University (IACUC NO. KW-220509-1). Mice were housed under temperature- and light-controlled conditions for 12 h daily and fed ad libitum. After induction of anesthesia with zoletyl 50, BLM (chemical industry, Tokyo, Japan) dissolved in 50 μl sterile saline was administered by direct intratracheal injection (1 mg/kg body weight). Control mice received an intratracheal dose of saline (50 μl). The body weight of the mice was monitored throughout the whole experimental period. Mice were given an intravenous (IV) injection of macrophage-derived CM (50 μg/kg) on days 7, 9, and 11 after BLM administration, and mice were sacrificed 14 days after BLM administration. For recombinant human CXCL11 (R&D SYSTEMS, Minneapolis, Minnesota, USA) treatment, the mice with fibrosis induction received an IV injection of CXCL11 (2 μg/mouse) on days 10, 12, 13, and 15 after BLM administration. Mice were sacrificed 17 days after BLM administration, and lung tissues were collected for the evaluation for PF.

### Lung digestion

Mice were euthanized and perfused with 5 ml PBS, which was followed by intratracheal instillation of 1 ml dissociation medium containing 0.8 U/ml collagenase/dispase (Roche) and 6 µg DNase (BioLabs, Massachusetts, USA) in RPMI 1640 medium. The lung tissue was minced to homogeneity using a razor blade, transferred to a 50 ml tube containing 3 ml of dissociation medium, and incubated for 30 min at 37 °C on a shaking platform. Next, 10 ml of cold PBS was added to the digested tissue, which was then filtered through a 70–μm filter and pelleted by centrifugation at 1200 rpm for 3 min at room temperature. The cells were then resuspended three times in PBS/1% fetal bovine serum (FBS) after red blood cell (RBC) removal using RBC lysis buffer. Macrophage polarization was analyzed by F4/80, CD80, and CD206 staining. Detailed information on the primary antibodies utilized is summarized in Supplementary Table 1.

### Cytokine array

Cytokine arrays of CM were analyzed using Proteome Profiler Human XL Cytokine Array Kit (R&D SYSTEMS) according to the manufacturer’s protocol. Intensity measurements were determined using an Image Lab 5.2 program (Bio-Rad, Hercules, CA, USA).

### Isolation of primary mouse bone marrow-derived macrophages (BMDM)

Bone marrow cells were isolated from tibias and femurs of C57BL/6 male mice. Cells were collected in a 5-ml round-bottom tube with a cell strainer cap (Falcon, San Jose, CA, USA) via flushing the bone marrow with DPBS (Hyclone, Logan, UT, USA) added to 2% FBS (Thermo). Cells were centrifuged at 500×*g*, washed with DPBS added to 2% FBS, and incubated with red blood cell (RBC) lysis solution (BioLegend, San Diego, CA, USA) for 5 min at 4 °C. After being washed with DPBS added to 2% FBS, collected cells were seeded for 7 days with 25 ng/ml macrophage colony-stimulating factor (M-CSF; PeproTech, Rocky Hill, NJ, USA) into RPMI medium (Thermo) supplemented with 10% FBS and 1% penicillin/streptomycin (Hyclone) incubated at 37 °C under a 5% CO_2_ atmosphere. The attached cells are BMDM.

### Flow cytometry

BMDMs were detached with 0.25% trypsin and centrifuged at 500×*g* for 5 min at 4 °C. The supernatant was removed, and the cell pellet was resuspended three times with 0.1% BSA (Thermo) in 1 × DPBS. Differentiation into macrophages was analyzed by CD45, CD11b, and F4/80 staining on a Beckman Coulter flow cytometer (Beckman Coulter, Brea, CA, USA) equipped with CyExpert software. The detailed information for primary antibodies is summarized in Supplementary Table 1.

### Immunohistochemistry

Lung tissues were paraffin-embedded, sectioned, deparaffinized, and rehydrated. The slides were subjected to hydration using an automated antigen retrieval of a citrate buffer (0.01 M sodium citrate, pH 6) before immunostaining. Endogenous peroxidase activity was then blocked by treatment with DAKO Real Peroxidase Blocking Solution for 20 min at room temperature (RT), followed by blocking with 10% normal goat serum for 1 h at RT. After incubation of the primary antibody in a blocking buffer overnight at 4 °C, fluorescently conjugated secondary antibodies were applied for 1 h at RT in the dark. Slides were washed with PBST (0.1% Tween), covered with Fluoroshield™ mounting medium containing 4′, 6-diamidino-2-phenylindole (DAPI), and captured with a fluorescence microscope (Olympus, Tokyo, Japan). At least three mice were used in each group, and data were obtained from at least three fields. The detailed information for primary antibodies is summarized in Supplementary Table 1.

### Histological examination

Paraffin sections of lung tissue were sequentially rehydrated with ethanol through a gradual concentration series. Sections were counterstained for nuclei using hematoxylin (BBC Biochemicals, Mt. Vernon, WA, USA) and cytoplasm by exposure to eosin and mounted with Permount mounting medium (Fisher Scientific). Sirius Red staining (Abcam, Cambridge, MA, USA) and Masson's trichrome (Empire Genomics, Buffalo, NY, USA) were performed according to the manufacturer's instructions. For histologic quantification, we used the Ashcroft score in a blinded fashion. The Ashcroft score ranges from 0 to 8, with 0 to 1 indicating no fibrosis, 2 to 3 indicating minimal fibrosis, 4 to 5 indicating moderate fibrosis, and 6 to 8 indicating severe fibrosis.

### IL-4-induced in vitro anti-inflammation model

BMDMs were seeded at 2.5 × 10^5^ cells/well in 12-well plates. The next day, 20 ng/ml IL-4 (R&D SYSTEMS) was treated for 12 h to induce M2 polarization in BMDM. At 12 h after IL-4 treatment, 100 ng/ml CXCL11 was treated for 6 h to induce M1 polarization. To induce M1 polarization, 100 ng/ml LPS (Sigma-Aldrich) was treated as a control. Inhibitors were treated 2 h before CXCL11. U0126 (MEK 1/2 inhibitor, 25 µM) and Wortmannin (AKT inhibitor, 2.5 nM) were purchased from Cell Signaling (Danvers, MA, USA). SB203580 (p38 inhibitor, 20 µM) was purchased from Selleck Chemicals (Houston, TX, USA). JSH-23 (NF-κB inhibitor, 5 µM) was purchased from MedChemExpress (Monmouth Junction, NJ, USA).

### Western blotting

Tissues and cells were lysed in a lysis buffer of PRO-PREP (iNtRON, Seongnam, Republic of Korea) solution and 1Xphosphatase inhibitor (Roche Applied Science, Indianapolis, IN, USA). The protein samples (10 μg/lane) were then separated by 8–15% SDS-PAGE, transferred onto a nitrocellulose membrane (Bio-Rad), and blocked with 5% skim milk (Bio-Rad) in TBS (Bio-Rad) containing 0.1% Tween 20 (Sigma-Aldrich). After blocking, membranes were subjected to Western blotting with appropriate primary antibodies overnight at 4 °C. The following day, membranes were incubated with a secondary antibody in 5% skim milk for 1 h at room temperature. The signals were developed using an ECL Western blotting substrate kit (Bio-Rad) and detected using Chemi-doc XRS + (Bio-Rad) with Image Lab 5.2 software (Bio-Rad). The detailed information for primary antibodies is summarized in Supplementary Table 2.

### RNA extraction and real-time RT-PCR

According to the manufacturer’s protocols, total RNA was extracted from the BMDMs using a TRIzol reagent (Ambion, Carlsbad, CA, USA). One microgram of total cellular RNA was subjected to RT using M-MLV (Promega, Madison, WI, USA) reverse transcriptase with random primers and oligo dT for cDNA synthesis. Synthesized cDNA was used for PCR with specific gene primers at appropriate cycle numbers and annealing temperatures (Supplementary Table 3). The PCR products were subjected to electrophoresis. Real-time RT-PCR was performed using SYBR Green Dye (Bio-Rad, Waltham, MA, USA) to quantify expression levels. A standard curve of cycle thresholds for several serial dilutions of a cDNA sample was established and used to calculate each gene’s relative abundance to compare transcript levels among samples. Values were then normalized to the relative amounts of *rPL7* cDNA.

### Statistics

All values are presented as mean ± standard deviation (SD). Student's *t*-test was used for comparisons between two groups, and one-way ANOVA analysis was used for comparisons between two or more groups. A *P*-value of less than 0.05 was considered statistically significant. Statistical analysis was performed using GraphPad Prism v.9 software (GraphPad Software, La Jolla, CA, USA).

## Supplementary Information

Additional file 1.

## Data Availability

The main article and supplemental information contain all the data related to this investigation.

## Abbreviations

AEC: Alveolar epithelial cells
BMDM: Bone marrow-derived macrophage
BrdU: Bromodeoxyuridine
CCR8: CC chemokine receptor 8
CM: Conditioned medium
COL1A1: Collagen, type 1, alpha 1
COX2: Cyclooxygenase 2
CXCL9: Chemokine ligand 9
CXCL11: C-x-c motif chemokine ligand 11
ccRCC: Clear cell renal cell carcinoma
ECM: Extracellular matrix
HE: Hematoxylin and eosin
hiPSC: Human induced pluripotent stem cells
iNOS: Inducible nitric oxide synthase
IV: Intravenously
(IFN)-γ: Interferon-γ
IL-4: Interleukin-4
LF: Liver fibrosis
LPS: Lipopolysaccharide
MAPK: Mitogen-activated protein kinase
M-CSF: Macrophage colony-stimulating factor
MEK: Mitogen-activated protein kinase kinase
miRs: MicroRNAs
PF: Pulmonary fibrosis
pERK: Phosphorylated extracellular signaling-related kinase
pAKT: Phosphorylated protein kinase B
PTX3: Pentraxin 3
SP-C: Surfactant protein C
TME: Tumor microenvironment
TGF-β1: Transforming growth factor β1
TNFα: Tumor necrosis factor α
αSMA: α-Smooth muscle actin
