# Supplementary material for: CXCL11 reprograms M2-biased macrophage polarization to alleviate pulmonary fibrosis in mice
Source: Cell Biosci. 2024 Nov 15;14:140. doi: 10.1186/s13578-024-01320-7 (PMC11566568; doi:10.1186/s13578-024-01320-7)
Supplement: Supplementary file 1 — Additional file 1. [file 13578_2024_1320_MOESM1_ESM.docx]

**Supplementary Information for**

CXCL11 reprograms M2-biased Macrophage Polarization to Alleviate Pulmonary Fibrosis in Mice

Ji-Young Kim^1^, Dong-Wook Cho^1^, Jung-Yun Choi^1^, Suji Jeong^1^, Minje Kang^1^, Woo Jin Kim^1^, In-Sun Hong^2^, Haengseok Song^3^, Heesoon Chang^4^, Se-Ran Yang^5^, Seung-Joon Lee^1^, Mira Park^3,†^, Seok-Ho Hong^1,4,†^

^1^Department of Internal Medicine, School of Medicine, Kangwon National University, Chuncheon, Republic of Korea

^2^Department of Health Sciences and Technology, GAIHST, Gachon University, Incheon, Republic of Korea

^3^Department of Biomedical Science, CHA University, Seongnam, Gyeonggi, Republic of Korea

^4^KW-Bio Co., Ltd, Chuncheon, Republic of Korea

^5^Department of Thoracic and Cardiovascular Surgery, School of Medicine, Kangwon National University, Chuncheon, Republic of Korea

**This Supplementary Material includes**:

**Supplementary Table 1**. List of antibodies for flow cytometry and IHC

**Supplementary Table 2**. List of antibodies for Western blotting

**Supplementary Table 3**. Primer sequences for RT-PCR and real-time RT-PCR

**Supplementary Figure 1**. RT-PCR on lung tissue from mice

**Supplementary Table 1. List of antibodies for flow cytometry and IHC**

| Antibody | Host | Supplier (Cat. No) | Dilution factor | Use for |
| --- | --- | --- | --- | --- |
| CD45 | Rat | BD Biosciences (559864) | 1:200 | Flow cytometry |
| CD11b | Rat | BD Biosciences (557396) | 1:200 | Flow cytometry |
| F4/80 | Rat | BD Biosciences (565410) | 1:200 | Flow cytometry |
| CD80 | Hamster | BD Biosciences (560016) | 1:200 | Flow cytometry |
| CD206 | Rat | Biolegend (141704) | 1:200 | Flow cytometry |
| iNOS | Rabbit | Novus (NB300-605) | 1:400 | IHC |
| CD206 | Rabbit | Abcam (ab64693) | 1:800 | IHC |
| Collagen1 | Rabbit | Abcam (ab21286) | 1:500 | IHC |
| αSMA | Rabbit | Abcam (ab124964) | 1:500 | IHC |
| BrdU | Rat | Abcam (ab6326) | 1:500 | IHC |
| F4/80 | Rat | Bio-rad(MCA497) | 1:400 | IHC |
| SP-C | Rabbit | Abcam (ab40879) | 1:800 | IHC |

**Supplementary Table 2. List of antibodies for Western blotting**

| Antibody | Host | Supplier (Cat. No) | Dilution factor | Use for |
| --- | --- | --- | --- | --- |
| p-AKT | Rabbit | Cell signaling (#9271) | 1:1000 | Western blotting |
| AKT | Rabbit | Cell signaling (#9272) | 1:1000 | Western blotting |
| p-p65 | Rabbit | Abcam (ab89299) | 1:1000 | Western blotting |
| p65 | Rabbit | Abcam (ab16502) | 1:1000 | Western blotting |
| p-ERK1/2 | Rabbit | Cell signaling (#9101) | 1:1000 | Western blotting |
| ERK1/2 | Rabbit | Cell signaling (#9102) | 1:1000 | Western blotting |
| COX2 | Rabbit | Abcam (ab15191) | 1:1000 | Western blotting |
| iNOS | Rabbit | Novus (NB300-605) | 1:1000 | Western blotting |
| ARG1 | Rabbit | Abcam (ab124917) | 1:1000 | Western blotting |
| CD206 | Rabbit | Abcam (ab64693) | 1:1000 | Western blotting |
| GAPDH | Rabbit | Cell signaling (#2118) | 1:3000 | Western blotting |
| Col1a1 | Mouse | Santa Cruz (sc-293182) | 1:500 | Western blotting |
| αSMA | Mouse | Santa Cruz (sc-293182) | 1:500 | Western blotting |
| TGFβ1 | Rabbit | Abcam (ab179695) | 1:1000 | Western blotting |
| SPC | Rabbit | Abcam (ab40879) | 1:1000 | Western blotting |
| AGER | Goat | R&D (AF1145) | 1:1000 | Western blotting |

**Supplementary Table 3. Primer sequences for RT-PCR and real-time RT-PCR**

| Gene |  | Sequence (5’-3’) | Size (bp) |
| --- | --- | --- | --- |
| *iNos* | F | TTCACCCAGTTGTGCATCGACCTA | 162 |
|  | R | TCCATGGTCACCTCCAACACAAGA |  |
| *Socs3* | F | CTTTGTAGACTTCACGGCTGCC | 232 |
|  | R | GGGAAACTTGCTGTGGGTGA |  |
| *Arg1* | F | AACACGGCAGTGGCTTTAACC | 132 |
|  | R | GGTTTTCATGTGGCGCATTC |  |
| *Mrc1* | F | CTGGATTGGACTCAACAGTCTGA | 161 |
|  | R | CCAGATTTTCCCACTTGGC |  |
| *rPL7* | F | TCAATGGAGTAAGCCCAAAG | 246 |
|  | R | CAAGAGACCGAGCAATCAAG |  |
| *IL6* | F | AGCCCTGAGAAAGGAGACAT | 175 |
|  | R | TGGAAGGTTCAGGTTGTTTT |  |
| *IL8* | F | GTGCAGTTTTGCCAAGGAGT | 187 |
|  | R | CTCTGCACCCAGTTTTCCTT |  |
| *TNFα* | F | AACCTCCTCTCTGCCATCAA | 184 |
|  | R | CCAAAGTAGACCTGCCCAGA |  |
| *CD206* | F | CACAAGCGCTGCGTGGAT | 95 |
|  | R | TTCGGACACCCATCGGAATTT |  |
| *IL10* | F | TACGGCGCTGTCATCGATTT | 190 |
|  | R | TAGAGTCGCCACCCTGATGT |  |
| *CD163* | F | ATGAAGATGCTGGCGTGACA | 68 |
|  | R | GGCTGCCTCCACCTCTAAGT |  |

F; Forward, R; Reverse


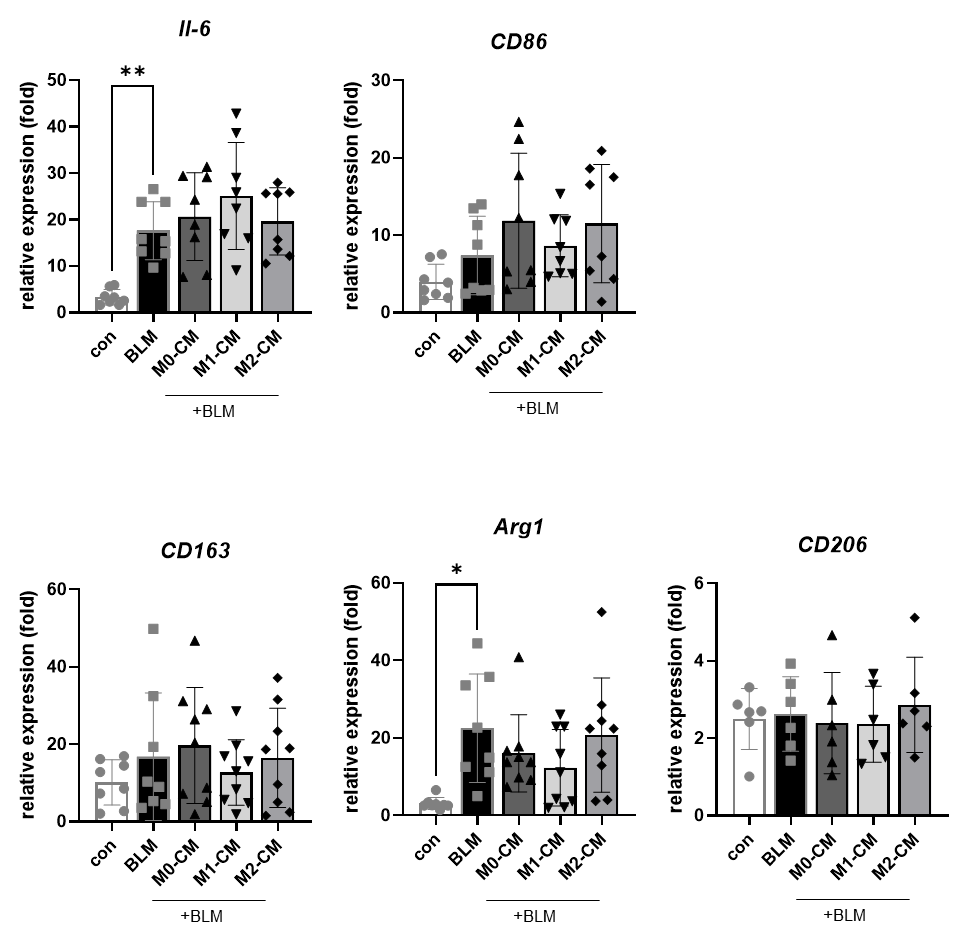
**Supplementary Figure 1. RT-PCR on lung tissue from mice**
